# Filaggrin Gene Mutation c.3321delA Is Associated with Various Clinical Features of Atopic Dermatitis in the Chinese Han Population

**DOI:** 10.1371/journal.pone.0098235

**Published:** 2014-05-23

**Authors:** Li Meng, Li Wang, Huayang Tang, Xianfa Tang, Xiaoyun Jiang, Jinhua Zhao, Jing Gao, Bing Li, Xuhui Fu, Yan Chen, Weiyi Yao, Wenying Zhan, Bo Wu, Dawei Duan, Changbing Shen, Hui Cheng, Xianbo Zuo, Sen Yang, Liangdan Sun, Xuejun Zhang

**Affiliations:** 1 Institute of Dermatology and Department of Dermatology, No.1 Hospital, Anhui Medical University, Hefei, Anhui, China; 2 Key Laboratory of Dermatology, Anhui Medical University, Ministry of Education, China, Hefei, Anhui, China; 3 State Key Laboratory Incubation Base of Dermatology, Anhui Medical University, Hefei, Anhui, China; 4 Department of Dermatology at No.2 Hospital, Anhui Medical University, Hefei, Anhui, China; 5 Department of Dermatology, Huashan Hospital of Fudan University, Shanghai, China; Kunming Institute of Zoology, Chinese Academy of Sciences, China

## Abstract

**Background:**

We confirmed that the filaggrin gene mutation c.3321delA is associated with atopic dermatitis in our previous genome wide association study of the Chinese Han population. c.3321delA is the most common filaggrin gene mutation in Chinese atopic dermatitis patients but is not present in European populations.

**Objective:**

To investigate the genetic model for the c.3321delA mutation and to determine the correlation between c.3321delA and atopic dermatitis clinical phenotypes in the Chinese Han population.

**Method:**

The filaggrin gene mutation c.3321delA was sequenced in 1,080 atopic dermatitis patients and 908 controls from the Chinese population. The χ^2^ test, ANOVA,nonparametric tests and logistic regression were used to investigate the relationship between the c.3321delA genotype and atopic dermatitis clinical phenotypes in the Chinese Han population.

**Results:**

Analyses of the genetic model revealed that the additive model best described the c.3321delA mutation (*P* = 3.09E-11, OR = 3.43, 95%CI = 2.38–4.96). Stratified analyses showed that the c.3321delA allele frequency distribution is significantly associated with concomitant skin xerosis (*P* = 1.68E-03, OR = 2.13,95%CI = 1.32–3.46), palmar hyperlinearity (*P* = 3.64E-17, OR = 4.0,95%CI = 2.86–5.70), white dermatographism (*P* = 4.25E-03, OR = 1.82,95%CI = 1.22–2.71), food intolerance (*P* = 1.51E-03, OR = 1.76,95%CI = 1.23–2.50) and disease severity ( *P* = 9.67E-05).

**Conclusion:**

Our study indicates that the filaggrin gene mutation c.3321delA is associated with clinical phenotypes of atopic dermatitis in the Chinese Han population, which might help us gain a better understanding on the pathogenesis of atopic dermatitis.

## Introduction

Atopic dermatitis (AD) has long been recognized as a complex trait, wherein multiple genes and environmental stimuli contribute to disease manifestation. To date, 81 genes have been implicated in over 100 published reports on AD genetic association studies, and 46 of these genes have demonstrated at least one positive association with AD. Of these genes, filaggrin gene (*FLG*) is the most consistently replicated gene, appearing in 20 reports [1]. Filaggrin, also known as filament-aggregating protein, plays a major role in the epidermal barrier function. To date, approximately 60 loss-of-function *FLG* mutations have been identified in European and Asian populations [2]. All of the mutations that are predicted to cause loss of function, including nonsense mutations as well as out-of-frame insertions or deletions, are specific to certain ethnic groups, with distinct profiles observed in the European and Asian populations that have been well studied [3].

AD, with a prevalence of 1.4 to 22.3% worldwide [4], has a complex clinical phenotype strongly associated with food allergies, asthma, and allergic rhinitis (AR) in a patient's life (i.e., the atopic march) [5]. Over the last decade, numerous association studies on *FLG* mutations and AD-associated phenotypes have been conducted. The majority of the studies focused on combined *FLG* mutations, and only a few studies referred to single mutation. c.3321delA is an Asian-specific *FLG* mutation that has been described in Chinese, Japanese, Korean and Singaporean populations [6]. c.3321delA is the most common *FLG* mutation in the Chinese population; however, it is not found in European populations. In our previous Genome Wide Association Study (GWAS) of AD, we identified the *FLG* variant rs3126085 that correlates with c.3321delA. In this study, we investigated the genetic model for c.3321delA, the genotype–phenotype correlation between c.3321delA and AD in the Chinese population. This study employs the largest sample size of all of the genotype-phenotype correlation studies of AD to date.

## Materials and Methods

### Patients and controls

A total of 1,080 AD patients and 908 controls were enrolled in this study **(** **Table 1** **)**. All samples were from the Chinese Han population and were used in our previous GWAS. AD patients meeting the Hanifin-Rajka diagnostic criteria [7] were recruited from the No. 1 Hospital of Anhui Medical University and the Xinhua Hospital affiliated with the Shanghai Jiaotong University School of Medicine in China. Physician specialists collected clinical data from the affected individuals through a full clinical checkup. Additional demographic information was collected from the cases and controls through a structured questionnaire. The disease severity was evaluated using the objective SCORing Atopic Dermatitis (SCORAD) index [8], which categorizes patients as mild (≤15 points), moderate (15–40 points) and severe (>40 points). Patients were considered to have food intolerance either evaluated by allergen test of venous blood samples or patients'self-reports. All controls were clinically assessed to be without AD or other atopic diseases, a family history of atopic diseases (including first-, second- and third-degree relatives) or ichthyosis vulgaris (IV). All participants provided written informed consent. The study was approved by the Institutional Ethics Committee of Anhui Medical University and was conducted according to the Declaration of Helsinki principles.

**Table 1:** The clinical characteristics of 1,080 cases.

Phenotype	Patients
Male (%)	629(58.24%)
Female (%)	451(41.76%)
Age (years), mean±SD (range)	5.14±6.42(0.5–58)
Age of onset (years), mean±SD (range)	1.03±3.00(0.02–37)
Early age of onset (≤2 years) (%) (n*)	999(92.59%)(1,079)
AD with asthma (%) (n*)	246(22.82%)(1,078)
AD with allergic rhinitis (%) (n*)	344(32.12%)(1,071)
AD with xerosis (%) (n*)	812(75.19%)(1,080)
AD with IV (%) (n*)	155(14.35%)(1,080)
AD with palmar hyperlinearity (%) (n*)	237(22.11%)(1,072)
AD with keratosis pilaris (%) (n*)	134(12.51%)(1,071)
AD with orbital darkening (%) (n*)	89(8.27%)(1,076)
AD with food intolerance (%) (n*)	407(42.66%)(954)
AD with white dermatographism (%) (n*)	158(14.65%)(1,080)
Elevated total IgE (>100 IU/ml) (%) (n*)	575(66.45%)(816)
Mild AD (objective SCORAD≤15) (%) (n*)	179(16.57%)(1,080)
Moderate AD (15< objective SCORAD≤40) (%) (n*)	764(70.74%)(1,080)
Severe AD (objective SCORAD>40) (%) (n*)	137(12.69%)(1,080)

### Statistical analyses

c.3321delA genotyping adhered to quality control standards, with a call rate >95% and meeting Hardy–Weinberg equilibrium (*P*>0.01) in the controls. The c.3321delA allelic and genotypic frequencies were compared between the AD patients and controls using the χ^2^ test with 2×2 and 2×3 contingency tables (SPSS 10.0, Statistical Program for Social Sciences, Illinois). The Fisher's exact test was used to compare the variable frequencies when the expected count was less than 5. Stratified analyses were performed to examine the relation between c.3321delA and the AD phenotypes. *P*<0.05 (two-tailed) was considered significant. The genetic models (dominant, recessive and additive models) were calculated for c.3321delA using logistic regression. To assess the effect of c.3321delA on the age of onset and disease severity, quantitative trait locus(QTL)analyses were performed in cases using ANOVA and nonparametric tests.

## Results

### Characteristics of the study subjects

The clinical characteristics of 1,080 patients (629 male and 451 female with a mean age of 5.14±6.42 years), including age of onset, AD with asthma, AD with AR, total IgE and AD severity, are summarized in Table 1. The 908 controls (629 male and 451 female) have a mean age of 16.35±9.32 years.

### The association of AD with c.3321delA

The c.3321delA *FLG* mutation was significantly associated with AD (*P* = 3.09 E-12,OR = 3.43, 95%CI = 2.38–4.96; *P*_genotype_ = 1.75E-11, OR = 2.93, 95%CI = 2.00–4.28). We further evaluated the homozygous and heterozygous odds ratio (OR_hom_/OR_het_) for c.3321delA in the cases and controls. Using allele A as the reference allele, the OR_het_ estimate of c.3321delA was 2.93 (95% CI = 2.00–4.28); however, the OR_hom_ estimates could not be calculated because no controls were homozygous for c.3321delA **(** **Table 2** **)**. Overall, the genetic model analysis revealed that the additive model best described the association of c.3321delA with AD (*P* = 3.09E-11, OR = 3.43, 95%CI = 2.38–4.96).

**Table 2:** Genotype of c.3321delA in 1,080 cases and 908 control.

	Cases (n = 1080)	Controls (n = 908)	OR (95% CI)	*P*
**Genotype**				
AA	949(87.87%)	871(95.93%)	Reference	
Aa	118(10.93%)	37(4.07%)	2.93(2.00–4.28)	1.75E-11
aa	13(1.20%)	0(0%)	NA	
**Recessive model**				
aa/(Aa+AA)	13/1067	0/908	NA	9.10E-04
**Dominant model**				
(aa+Aa)/AA	131/949	37/871	3.25(2.23–4.73)	1.26E-10
**Additive model**				
aa/Aa/AA	13/118/949	0/37/871	3.43(2.38–4.96)	3.09E-11

### Clinical phenotype stratification analyses

We also assessed the association between c.3321delA and AD phenotypes **(** **Table 3** **)** using stratified analyses in the cases. Significant associations were observed between c.3321delA and concomitant skin xerosis (*P* = 1.68E-03, OR = 2.13, 95%CI = 1.32–3.46), IV (*P* = 2.17E-02, OR = 1.63, 95%CI = 1.07–2.49), palmar hyperlinearity (*P* = 3.64E-17, OR = 4.03, 95%CI = 2.86–5.70), keratosis pilaris (*P* = 1.72E-02, OR = 1.70, 95%CI = 1.09–2.64), white dermatographism (*P* = 4.25E-03, OR = 1.82, 95%CI = 1.22–2.71) and food intolerance (*P* = 1.51E-03, OR = 1.76, 95%CI = 1.23–2.50) **(** **Table 3** **)**. In the QTL analysis, we found that c.3321delA was associated with disease severity (*P* = 9.67E-05). The c.3321delA homozygous and heterozygous patients displayed a significantly increased average SCORAD score (32.87 and 30.79, respectively) compared with the patients with a wild-type genotype (25.73) **(** **Table 4** **)**. The patients harboring c.3321delA (homozygous and heterozygous) displayed a trend of earlier age of onset (0.16 and 0.81 years, respectively) compared with the wild-type genotype (1.07 years), which although displayed no statistical significance but showed a trend among three groups (*P* = 0.056) **(** **Table 4** **)**. We observed that the c.3321delA allele frequencies in AD patients without asthma or AR were slightly higher than in patients with asthma or AR, but the differences were not statistically significant (all *P*>0.05) **(** **Table 3** **)**. In stratified analyses, c.3321delA was not associated with other phenotypes of AD, including early age of onset, elevated total serum IgE levels and orbital darkening ( *P*>0.05) **(** **Table 3** **)**.

**Table 3:** The association between c.3321delA and clinical phenotypes in AD.

Clinical phenotypes	Allele frequencies		*P*	OR	95%CI
	3321delA	A			
Early age of onset (≤2 years)	0.0691	0.9309	1.24E-01	1.90	0.83–4.38
Late age of onset (>2 years)	0.0375	0.9625			
AD with asthma	0.0650	0.9350	8.60E-01	0.96	0.64–1.45
AD without asthma	0.0673	0.9327			
AD with AR	0.0581	0.9419	2.97E-01	0.82	0.56–1.19
AD without AR	0.0702	0.9299			
AD with elevated IgE	0.0740	0.9260	6.59E-01	1.10	0.77–1.56
AD with normal IgE	0.0685	0.9315			
AD with Xerosis	0.0764	0.9237	1.68E-03	2.13	1.32–3.46
AD without Xerosis	0.0373	0.9627			
AD with IV	0.0968	0.9032	2.17E-02	1.63	1.07–2.49
AD without IV	0.0616	0.9384			
AD with Palmar hyperlinearity	0.1519	0.8481	3.64E-17	4.03	2.86–5.70
AD without Palmar hyperlinearity	0.0425	0.9575			
AD with Keratosis pilaris	0.1007	0.8993	1.72E-02	1.70	1.09–2.64
AD without Keratosis pilaris	0.0619	0.9381			
AD with Orbital darkening	0.0506	0.9494	3.62E-01	0.73	0.36–1.45
AD without Orbital darkening	0.0684	0.9316			
AD with food intolerance	0.0842	0.9158	1.51E-03	1.76	1.23–2.50
AD with food tolerance	0.0496	0.9504			
AD with White dermatographism	0.1107	0.8893	4.25E-03	1.82	1.22–2.71
AD without White dermatographism	0.0642	0.9358			

**Table 4:** Association between genotype of c.3321delA and SCORAD and age of onset in AD.

Genotypes	AA	Aa	aa	*P*
Patients (n)	927	118	13	
Objective SCORAD (median)	25.73(24.00)	30.79(29.30)	32.87(32.00)	9.67E-05
Age of onset (years) (median)	1.07 (0.17)	0.81 (0.17)	0.16 (0.083)	0.056

### The relationship between age of onset and c.3321delA-associated phenotypes of AD

In order to explore whether patients with earlier onset tend to present the phenotypes associated with c.3321delA or whether patients with mutation related phenotypes display a trend of earlier onset, we divided the patients into two groups (early age of onset and late age of onset), calculated these phenotypes' prevalence of the two groups, and compared the phenotype-distribution difference using the χ^2^ test. We observed that the prevalence of AD concomitant with IV in the early age of onset group was significant lower than late age of onset group (13.37% vs 27.27%, *P* = 0.004). On the other hand, AD concomitant with IV displayed a trend of later onset than without IV (1.56 years vs 0.91 years, *P* = 0.026), as well as AD concomitant with keratosis pilaris also had a trend of later onset than without keratosis pilaris (1.69 years vs 0.91 years, *P* = 0.034) (**Table 5**). There was no significant difference between age of onset and phenotypes (xerosis, palmar hyperlinearity, food intolerance and white dermatographism) **(** **Table 5** **)**.

**Table 5:** The relationship between age of onset and c.3321delA related phenotypes in AD.

c.3321delA related phenotypes of AD	Early age of onset	Late age of onset	P*	OR(95%CI)	Cases(n)	Mean onset age(SD)	*P^#^*
With xerosis	750(75.53%)	56(72.73%)	0.953	0.99(0.69–1.42)	812	0.95(3.10)	0.256
Without xerosis	243(24.47%)	21(27.27%)			266	1.19(2.59)	
With IV	132(13.37%)	21(27.27%)	0.004	2.09(1.25–3.50)	154	1.56(3.35)	0.026
Without IV	855(86.63%)	56(72.73%)			917	0.91(2.91)	
With palmar hyperlinearity	221(22.39%)	16(20.78%)	0.872	0.96(0.55–1.67)	237	1.13(2.86)	0.468
Without palmar hyperlinearity	766(77.61%)	61(79.22%)			834	0.97(3.02)	
With keratosis pilaris	118(11.96%)	15(20.00%)	0.084	1.67(0.93–3.00)	134	1.69(4.12)	0.034
Without keratosis pilaris	869(88.04%)	60(30.00%)			937	0.91(2.77)	
With food intolerance	379(43.22%)	22(30.14%)	0.163	0.70(0.43–1.14)	404	0.97(2.90)	0.322
Without food intolerance	498(56.78%)	51(69.86%)			552	1.18(3.31)	
With white dermatographism	119(14.39%)	10(15.87%)	0.718	1.14(0.57–2.28)	131	1.38(3.43)	0.109
Without white dermatographism	708(85.61%)	53(84.13%)			763	0.93(2.94)	

P* value: calculated by χ^2^ test; *P^#^* value:calculated by T test;

## Discussion

In our previous GWAS, we confirmed that the *FLG* mutation c.3321delA is associated with AD in the Chinese Han population [9]. In the current study, our genotype–phenotype analyses of AD may aid in the investigation of various disease phenotypes and the identification of phenotype-specific genetic factors, thereby providing new insights into the pathogenesis of AD. Our findings indicate that c.3321delA significantly associates with various AD clinical phenotypes, including skin xerosis, IV, palmar hyperlinearity, keratosis pilaris, white dermatographism, food intolerance and disease severity.

The association of c.3321delA in our group was best described with an additive model that displayed a clear trend for increased disease risk in heterozygous and homozygous c.3321delA patients. Our analysis comparing AD severity (measured by the objective SCORAD score) between the genotype groups in the QTL analysis showed that homozygous and heterozygous c.3321delA patients were more likely to have a more severe form of the disease, and this finding is consistent with the Singaporean study that showed that the combined null *FLG* genotype of 17 mutations detected in cases and controls were strongly associated with increased AD severity (permutation test *P* = 0.0063) [6]. However, the association was inconsistent in other studies [2], [10], [11] in the Chinese Han population, all of which were assessing compound genotypes of *FLG* including c.3321delA with smaller samples. *FLG* mutations predict dose-dependent alterations in epidermal permeability barrier function [12], and our results confirmed that the *FLG* null mutations might serve as an indicator of severe disease phenotypes.

Several studies indicate that *FLG* mutations have an effect on the age of onset of AD such that individuals carrying *FLG* mutations (R501X, 2282del4, R2447X or S3247x) can lead to early-onset (age of onset ≤2 years) AD that persisits well into adulthood [13]–[16]. Moreover, Ma et al. reported that c.3321delA was associated with early-onset of AD in Northern Chinese patients (*P* = 0.020) [17]. However, in our study, no statistical significance was observed for the association between early-onset at AD and *FLG* mutation c.3321delA in stratified analysis (*P* = 1.24E-01), which may be attributed to the fact that the majority of AD cases begin early in life (age of onset ≤2 years). It's reported that *FLG* mutations were associated with much earlier age at onset for AD [11], [18], AD patients carrying *FLG* mutations were younger than those without *FLG* mutations. However, one study in China did not observe the association (*P* = 0.307) [2]. In our study, we observed that the average/median age of onset in AD tended to decrease among the three groups (wide-type, homozygous and heterozygous genotype) in the QTL analysis, but there were no statistical differences (*P* = 0.056), which may be due to the low proportion of AD cases with homozygous c.3321delA. Further analysis using larger sample sizes will be helpful for determining the effect of c.3321delA on age of onset.

Our data provide evidence for the association between c.3321delA and various AD phenotypes, including concomitant IV, palmar hyperlinearity, and keratosis pilaris (**Table 3**), consistent with previous studies [6], [10] regarding *FLG* compound mutations. A recent study in Northern China indicated that combined *FLG* variants were significantly associated with IV and palmar hyperlinearity; however, no association with keratosis pilaris was observed [2]. These results are attributed to the fact that AD has a well-recognized association with IV [19], [20] and that *FLG* is the pathogenic gene of IV; thus, AD patients from non-IV family trios have a low probability of carrying *FLG* mutations [10]. Hyperlinear palms and keratosis pilaris, the phenotypic characteristics of IV, have been previously reported to be strong clinical markers of *FLG*-null mutations [21]. Greater than 60% of patients carrying *FLG* mutations develop palmar hyperlinearity manifested as criss-cross hyperlinearity of the thenar eminence [22]. Hyperlinear palms,keratosis pilaris and IV were all skin barrer dysfunction disorders, and they have common pathogenesis with AD, thus they could represent good phenotypic indicators of *FLG*-null mutations and AD.

In this study, we first reported that c.3321delA was associated with AD coexistent skin xerosis in Asian populations. A similar study using a smaller sample in Northern China did not find this association [2]. But the German study has reported a strong association between combined *FLG* mutations and dry skin [21]. *FLG* gene number polymorphisms has been associated with the dry skin phenotype [23]. The loss or reduction of filaggrin expression disrupts barrier formation making filaggrin-deficient skin susceptible to increased transepidermal water loss and easy penetrated by environmental allergens, which can manifest as varying degrees of dry skin. The filaggrin degradation products, namely several amino acids (alanine, pyrrolidone carboxylic acid, and urocanoic acid) act as natural moisturizing factors (NMFs) in the stratum corneum. The hygroscopic NMFs are important for the maintenance of epidermal barrier hydration. NMFs are less abundant in dry skin [24], which becomes more pronounced with age [25] and changing seasons. Biochemical and immunological evidence indicate that profilaggrin and processed filaggrin are completely absent in patients carrying *FLG* mutations [26]. *FLG* deficiency or absence results in reduced NMFs and impaired epidermal barrier function, which likely contribute to the etiopathogenesis of AD and AD-associated skin xerosis. Therefore, our results are the first to suggest that *FLG* mutations may be associated with an individual's predisposition to skin xerosis in the context of AD.

We also were the first to report that c.3321delA was associated with AD coexistent white dermatographism using a large sample study of an Asian population, whereas a similar study using a smaller sample from Northern China did not observe the association [2]. White dermatographism expresses as local erythema followed by edema and a surrounding flare reaction caused by a stroke with a dull object. Dermatographism is likely considered to be caused by mechanicoimmunological stimulation of mast cells that release histamine. Mechanical trauma is thought to release an antigen that interacts with IgE-sensitized mast cells, which further release inflammatory mediators like histamine into the tissues. Filaggrin degradation can release histidine acting as putative ultraviolet photoprotector [27]. Our result indicated that *FLG* mutation c.3321delA might involve in the development of white dermatographism.

In addition, we found that c.3321delA was associated with food intolerance, consistent with the Southern China study [11] assessing compound genotypes for combined *FLG* variants and the study by Linneberg et al [28] assessing *FLG* mutations R501X or 2282del4. *FLG* mutations predispose individuals to AD with allergic sensitization [29]. Studies have indicated that the absorption of allergens through the skin of patients with *FLG* mutations may be a predisposing factor for the development of other allergic disorders [30]. The destruction of normal epidermal barrier function is considered a key event in allergic sensitization [31]. A damaged epidermal barrier allows allergens to penetrate into the skin easily and exposes the allergens to antigen-presenting cells, causing allergic sensitization. Except skin keratinizing epithelial tissue, filaggrin is also expressed in other keratinizing epithelia, such as the oral and nasal mucosa, and is also presumed to contribute to the oral epithelial barrier function. Since, allergen through oral can also cause allergy. Our results confirm that skin barrier defects due to *FLG* mutations play a crucial role in the pathogenesis of other allergic disorders, such as food sensitization.

The relationship among AD, asthma, AR and *FLG* mutations is complex. Common and varying genetic factors for AD and asthma have been reported in the Chinese Han population [32]. A large European cohort study demonstrated that *FLG* null mutations predispose to allergic phenotypes, such as asthma and AR involved in the atopic march only in the presence of eczema [33]. Several studies have shown that *FLG* mutations predispose to asthma but only in the context of prior eczema or AD and their families, which indicating that *FLG* mutations did not have an independent effect on asthma [34]–[36]. A study [17] in Northern China reported that c.3321delA was associated with AD-associated AR or asthma in stratified analysis (*P* = 0.035). However, a Polish study also found evidence for the association between combined *FLG* variants with asthma and AR, and an association between *FLG*-null variants and atopic asthma was also observed in individuals without AD or a history thereof [37]. In one study in Northern China, no association was observed between the combined *FLG* mutations and AD-associated asthma or AR; however, an association between the mutation K4671X and AD-associated AR was found [2]. An additional study in Southern China did not find an association between the combined *FLG* mutations and AD-associated asthma or AR. However, c.3321delA was found more frequently in AD patients without asthma than patients with asthma. In addition, a significant association between c.3321delA and AD patients with asthma was observed (*P* = 0.016) [11], implying that c.3321delA may serve as a protective factor for asthma that occurs in the context of AD. In this larger sample study, we observed that the c.3321delA allele frequencies in AD patients without asthma or AR were slightly increased compared with AD patients with asthma or AR, but the findings were not statistically significant (*P* = 0.86, OR = 0.96, 95%CI = 0.64–1.45 and *P* = 0.297, OR = 0.82, 95%CI = 0.56–1.19, respectively) **(** **Table 3** **)**. The negative association between *FLG* mutations and atopies found in our study may be attributed to sample bias, environmental factors and ethnic differences. The majority of our patients displayed an age of onset for asthma and AR well below the median age at onset. However, we confirmed the previous hypothesis that c.3321delA might be a protective factor for asthma. Additional studies are needed to clarify the relationship between *FLG* mutations and AD-associated respiratory allergic disorders.

We also performed an analysis regarding orbital darkening, but no significant difference was found, consistent with the Northern China study [2]. AD patients tend to have increased IgE levels. A study from Japan reported that c.3321delA is associated with elevated IgE levels [38], but we were unable to replicate that association in this study, which is consistent with the south China [11] and Polish [37] studies.Above all, according to our results, mutation c.3321delA was associated with several AD phenotypes, and AD patients with c.3321delA tended to have earlier age of onset, though the association is not significant. Since this, we performed further stratified analysis to explore the possible relationship between age of onset and these six AD phenotypes associated with c.3321delA. And we found that AD patients with late age of onset were more likely to accompany with IV, and AD patients with IV or keratosis pilaris tended to have later age of onset in AD. This may be due to the later predilection ages of IV and keratosis pilaris than that of AD.

Of course, there are some limitations in our study. Our study is limited by all cases and controls being of Chinese Han and it is possible that the *FLG* mutations may not exert the same effect in other races of China and other Asian countries. Our sample size was not large enough, so that most ( 92.59%) of our patients were early age of onset (≤2 years), and only 13 patients were homozygous, which may lead to minor bias. In addition, the phenotypes-genotypes association analysis in current study was just focused on AD patients, and all the results about phenotypes-genotypes relationship were in the presence of AD. In future study, we will perform the associated analyses of c.3321delA in the non-AD group, such as groups of IV, asthma and AR, et al.

In conclusion, our study confirmed that the *FLG* mutation c.3321delA was associated with AD under an additive genetic model in a large Chinese cohort. In addition, we observed a correlation between c.3321delA and various clinical features of AD, and we demonstrated that c.3321delA has an effect on these phenotypes in the context of AD. These findings may help to further define the role of *FLG* in AD susceptibility, thereby assisting in the categorization of various subtypes of the disease and building the foundation for genetic diagnosis and personalized treatment for patients with AD in the near future.
